# A survey of mosquito-borne and insect-specific viruses in hospitals and livestock markets in western Kenya

**DOI:** 10.1371/journal.pone.0252369

**Published:** 2021-05-28

**Authors:** Tatenda Chiuya, Daniel K. Masiga, Laura C. Falzon, Armanda D. S. Bastos, Eric M. Fèvre, Jandouwe Villinger

**Affiliations:** 1 International Centre of Insect Physiology and Ecology (*icipe*), Nairobi, Kenya; 2 Department of Zoology and Entomology, University of Pretoria, Pretoria, South Africa; 3 Institute of Infection, Veterinary and Ecological Sciences, University of Liverpool, Neston, United Kingdom; 4 International Livestock Research Institute, Nairobi, Kenya; CEA, FRANCE

## Abstract

*Aedes aegypti* and *Culex pipiens* complex mosquitoes are prolific vectors of arboviruses that are a global threat to human and animal health. Increased globalization and ease of travel have facilitated the worldwide dissemination of these mosquitoes and the viruses they transmit. To assess disease risk, we determined the frequency of arboviruses in western Kenyan counties bordering an area of high arboviral activity. In addition to pathogenic viruses, insect-specific flaviviruses (ISFs), some of which are thought to impair the transmission of specific pathogenic arboviruses, were also evaluated. We trapped mosquitoes in the short and long rainy seasons in 2018 and 2019 at livestock markets and hospitals. Mosquitoes were screened for dengue, chikungunya and other human pathogenic arboviruses, ISFs, and their blood-meal sources as determined by high-resolution melting analysis of (RT-)PCR products. Of 6,848 mosquitoes collected, 89% were trapped during the long rainy season, with *A*. *aegypti* (59%) and *Cx*. *pipiens* sensu lato (40%) being the most abundant. Most blood-fed mosquitoes were *Cx*. *pipiens* s.l. with blood-meals from humans, chicken, and sparrow (*Passer* sp.). We did not detect dengue or chikungunya viruses. However, one *Culex poicilipes* female was positive for Sindbis virus, 30 pools of *Ae*. *aegypti* had cell fusing agent virus (CFAV; infection rate (IR) = 1.27%, 95% CI = 0.87%-1.78%); 11 pools of *Ae*. *aegypti* had Aedes flavivirus (AeFV; IR = 0.43%, 95% CI = 0.23%-0.74%); and seven pools of *Cx*. *pipiens* s.l. (IR = 0.23%, 95% CI = 0.1%-0.45%) and one pool of *Culex annulioris* had Culex flavivirus. Sindbis virus, which causes febrile illness in humans, can complicate the diagnosis and prognosis of patients with fever. The presence of Sindbis virus in a single mosquito from a population of mosquitoes with ISFs calls for further investigation into the role ISFs may play in blocking transmission of other arboviruses in this region.

## Introduction

Mosquitoes of the genera *Culex* and *Aedes* are the major vectors of arboviruses, bridging the transmission of viruses from the sylvatic world to urban settings [1]. Mosquitoes of the *Culex pipiens* complex transmit West Nile and Sindbis viruses, and have been implicated in the transmission of Rift Valley fever virus [2]. West Nile virus, first documented in Uganda [3], causes self-limiting febrile illness, which in rare cases proceeds to a fatal meningoencephalitis, while Sindbis virus also causes a febrile illness associated with chronic arthritis in humans [1]. Passerine birds are the reservoir and amplifying hosts for both viruses, while mammals, when infected, are considered dead-end hosts [4–6]. *Aedes aegypti* transmits dengue, chikungunya, Zika, yellow fever, and Rift Valley fever viruses, which are endemic in East Africa, including Kenya [7].

The ability of these viruses to cause worldwide epidemics is of increasing concern due to intensified globalization and travel [8–10]. Globally, vaccines against arboviruses are either not available or have limited use, and treatment is usually palliative [8, 11]. In developing countries, inadequate diagnostic capacity for these viruses is an additional challenge, especially in areas where other causes of febrile disease, like malaria, are present [12].

Arboviral disease control is more likely to be successful when the vector species present, and their competence, is known. Residual spraying with insecticides and the use of insecticide-treated bed nets, have been successful in reducing malaria transmission, but have achieved less in reducing arbovirus transmission due to differences in the feeding and resting behaviour of anopheline and culicine mosquitoes [13]. The use of insect-specific flaviviruses (ISFs) that naturally infect *Aedes* and *Culex* mosquitoes as potential regulators against infection with pathogenic arboviruses via superinfection exclusion mechanisms has been suggested [14]. Replication of ISFs in co-infected cells is believed to be more efficient, thereby competitively suppressing the proliferation of pathogenic arboviruses [15]. However, such superinfection mechanisms may be restricted to specific arbovirus-ISF pairings.

Many *Aedes* and *Culex* mosquitoes are adapted to a domestic life cycle, breeding in man-made habitats and biting people indoors and outdoors. Some of their breeding sites include open septic tanks, bushy/grassy places, discarded tyres/cars, jars, drums, and any other open water containers [1, 16]. Studies in East Africa have demonstrated the presence of several arboviruses of public health importance [7, 17], but the links between human and livestock infections have not been explored. Therefore, in this study, we surveyed selected hospitals and livestock markets (LMs) in western Kenya for the presence of mosquito-borne viruses. Specifically, we investigated mosquito diversity and abundance associated with these settings, host-feeding preferences, and the frequency of arboviruses and ISFs within the mosquitoes. Additionally, we described the implementation of mosquito control strategies at hospital sites.

## Materials and methods

### Sampling sites selection

The study was carried out in the western Kenyan counties of Bungoma, Busia, and Kakamega, which border Uganda. This region occurs within the wider Lake Victoria basin of East Africa whose ecology is likely to support an abundant mosquito population. The selection of sampling sites is described elsewhere [18]. Briefly, 12 LMs and neighbouring hospitals, four in each of the three counties, were selected for an integrated surveillance program. The selection of the LMs was based on the size and catchment area, whereas selection of the hospitals was based on the number of outpatients and the type of hospital. Specifically, both public (Referral and sub-County) and private (Missionary) hospitals were included. Finally, logistical factors such as the distance to the field laboratory in Busia, were also taken into consideration.

For this survey, six hospitals (one public and one missionary hospital in each county) were originally selected for mosquito sampling. Factors impacting mosquito habitat, resting, and feeding behavior, such as hospital size and in/outpatient number, were considered in the selection process. Similarly, availability of mosquito habitats, resting places, proximity to human dwellings, and security for setting up mosquito traps were considered when selecting LMs in each county.

**Fig 1:**
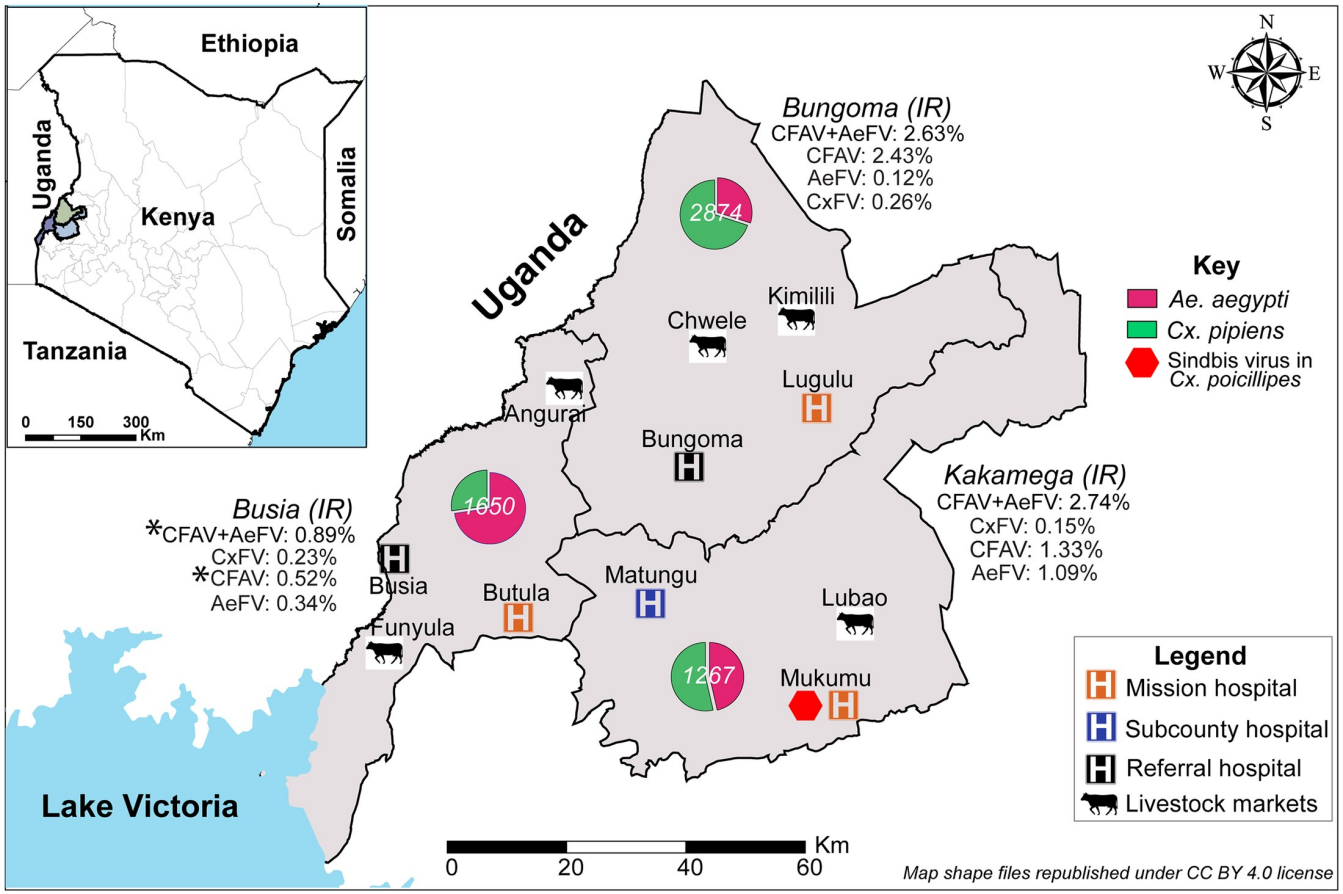
Sites where mosquito traps were set in the three counties in western Kenya. Pie charts of the relative abundance of *Ae*. *aegypti* and *Cx*. *pipiens* s.l. and their infection rates (IRs) with cell fusing agent virus (CFAV)/Aedes flavivirus (AeFV) and Culex flavivirus (CxFV), respectively, are shown for each county. Infection rates that were significantly lower in Busia than in the other two counties are indicated by asterisks. Water bodies, country and county boundary data were downloaded and re-published under CC BY 4.0 license from the World Resources Institute website (https://www.wri.org/resources/data_sets) [19]. The map was developed using QGIS software version 3.16.1 (https://www.qgis.org/en/site/) [20].

A pilot study was conducted in the short rainy season from 17 October 2018 to 7 December 2018 at six hospitals (Lugulu Missionary, Bungoma Referral, Busia Referral, Butula Missionary, Matungu sub-County, and Mukumu Missionary) and four LMs (Lubao, Angurai, Kimilili, and Chwele). Sampling in the long rainy season was done from 9 May to 26 June 2019 when mosquito habitat and density were expected to be high. The same six hospitals were sampled during the long rainy season; however, due to poor mosquito catches and logistical challenges experienced at some LMs during the short rainy season (pilot), where we were unable to adequately secure the trapping equipment despite the security measures put in place, only Lubao LM and an additional LM in Funyula were sampled. Fig 1 shows the locations of all the hospitals and LMs where mosquitoes were sampled in the short and long rainy seasons.

### Questionnaire on mosquito control at hospitals

Concurrently with the mosquito collection at hospitals during the long rainy season, a short questionnaire was administered to capture information about the methods implemented to control the breeding of mosquitoes and prevent them from biting patients and personnel at hospitals (S1 File). The questionnaire was administered by the same individual at all sites to the public health officers, medical superintendents, or hospital administrators. Direct observations on the presence of environmental features suited to the breeding and presence of mosquitoes were also recorded.

### Mosquito trapping and schedule

During the short rainy season (pilot), seven CDC light (John W. Hock Company, Gainesville, USA) and seven BG sentinel traps with a lure (Biogents, Regensburg, Germany) were set for one night and one day, respectively, at each site. All traps were baited with dry ice delivered from insulated dry ice containers. CDC light traps were set and run from 6:30 pm to 6:30 am the next day, while BG sentinel traps were run from 6:30 am to 6:15 pm. In the long rainy season, seven CDC light traps and seven BG sentinel traps were set for three consecutive nights and days, respectively, at the six hospitals. Due to security constraints experienced at the LMs the CDC and BG traps were set for two consecutive nights and days, respectively.

Traps in hospital settings were placed away from direct wind, foot traffic, and artificial lighting. Preferred locations for CDC traps were disused pit latrines, dilapidated buildings, broken-down vehicles, and uncovered septic tanks. In some instances, and following consultation with the hospital staff, traps were set in patient wards and consultation rooms. BG sentinel traps were placed in grassy or bushy locations of the hospital, away from direct sunlight and wind gusts. At LMs, CDC light traps were hung around the perimeter of the market and close to any surrounding homesteads, while BG sentinel traps were placed in grassy shaded places around the markets. Traps were set for a cumulative 231 trap days and 223 trap nights; these included 63 trap days and nights during the pilot phase conducted during the short rainy season, and 168 trap days and 160 trap nights in the long rainy season.

This study was nested within the Zoonoses in Livestock in Kenya (ZooLinK) project. The trapping of mosquitoes and interviews at hospitals were carried out under the approval of the International Livestock Research Institute (ILRI) Institutional Research Ethics Committee (IREC) under protocol number ILRI-IREC2017-08/2. ILRI IREC is registered and accredited by the National Commission for Science, Technology and Innovation (NACOSTI) in Kenya, and approved by the Federalwide Assurance for the Protection of Human Subjects in the USA. At each of the selected hospitals sampling was carried out with permission from the medical superintendent or administrative officer. In Kakamega County, Matungu sub-County Hospital replaced the planned sampling site at Kakamega Referral Hospital due to logistical and consent challenges. At LMs, the chairpersons of the two markets were informed of the planned exercise before sampling began.

### Storage of mosquitoes and identification

Mosquitoes were collected alive in the evening and early morning. They were anaesthetized with ethyl acetate, sorted to remove non-target insects and stored in cryovials in a nitrogen tank. They were shipped to the Martin Lüscher Emerging Infectious Disease (ML-EID) Laboratory at the International Centre of Insect Physiology and Ecology (*icipe*) in Nairobi, where they were morphologically identified on a chilled surface under a stereomicroscope with the aid of identification keys [21, 22]. Mosquitoes were grouped in pools of up to 25 mosquitoes per pool, according to the site, trap type, date of collection, sex, and species. Blood-fed mosquitoes were placed individually in Eppendorf tubes for blood-meal determination.

### Nucleic acid extraction

We homogenized mosquito pools and individual mosquito abdomens by mechanical disruption in 1.5-ml micro-centrifuge tubes with ten 2.0-mm zirconia/yttria stabilised zirconium oxide beads (Biospec, USA) using a Mini Bead Beater 16 (BioSpec, Bartlesville, USA) for 45–70 seconds at a frequency of 3,450 revolutions per minute. For blood-fed mosquitoes, the abdomens were separated from the rest of the body using sterile 10-μl pipette tips before processing. After homogenization, 410 μl of phosphate-buffered saline was added to each micro-centrifuge tube containing either a mosquito pool, or an engorged abdomen. The magnetic-based Magbio HighPrep^™^ Viral DNA/RNA Kit (Gaithersburg, USA) was used for rapid isolation of total nucleic acids. Initially, 200 μl of the homogenized sample was mixed with 528 μl of a lysis master-mix, 10 μl magnetic beads, and 10 μl proteinase K before proceeding with the rest of the protocol according to the manufacturer’ instructions. Total nucleic acid was eluted in 100 μl elution buffer. Dengue serotype 2 and Sindbis viruses cultured on Vero cell lines in a previous study were included as positive extraction controls in each extraction run. For both viruses, high (10^−2^) and low (10^−6^) viral titre controls were included in the extraction [23]. The extraction was carried out in a PCR enclosure and to minimize the chances of cross contamination, the controls were always extracted in the last runs with change of gloves and decontamination of pipettes with RNase AWAY^®^ (Molecular Bio-Products, New York, USA) between runs.

After nucleic acid extraction, 15 μl of the total RNA was subjected to cDNA synthesis using a High Capacity cDNA Reverse Transcription (RT) Kit (Life Technologies, USA). The 30-μl reaction mixtures contained 1X RT buffer, 4 mM dNTPs, 600 μM random hexamers [24], 2.5 U/μl reverse transcriptase enzyme, and 1U/μl RNAse inhibitor.

### Blood-meal analysis

We carried out blood-meal analysis on each individually extracted blood-fed mosquito to determine its vertebrate host. We used primers for cytochrome b (cyt *b*) and 16S rRNA markers to resolve the vertebrate host source of the blood-meals [25]. Total nucleic acid (1 μl) from each blood-fed mosquito was used as template in 10-μl PCRs containing 2 μl of 5X HOT FIREPol^®^ EvaGreen^®^ qPCR Mix (Solis BioDyne, Estonia) and 10 pmoles of each forward/reverse primer. Thermo-cycling and high-resolution melting (HRM) analysis were carried out in a Rotor-Gene Q real-time PCR thermo-cycler (Qiagen, Hilden Germany) as previously described [26]. DNA extracted from human, cattle, sheep, goat, pig, camel, and chicken samples served as positive controls in each of the runs. Rotor-Gene Q software 2.1.0 was used to select representative amplicons for post-PCR clean (Exo 1-rSAP combination, Biolabs, UK) and sequencing at Macrogen (The Netherlands).

### Molecular detection of viruses

Mosquito pools were screened for six genera (*Flavivirus*, *Alphavirus*, *Phlebovirus*, *Orthobunyavirus*, *Nairovirus*, and *Thogotovirus*) of arboviruses using a multiplex PCR that uses degenerate primers coupled with end-reaction high resolution melting analysis (PCR-HRM). Briefly, this test has a high analytical sensitivity and is able to detect viral nucleic acid in as low as 20–200 PFU/ml for flaviviruses and thogotoviruses, and 2–20 PFU/ml for alphaviruses and orthobunyaviruses [23]. While this test can distinctly detect 15 different arboviruses across the six genera of interest with analytical sensitivity comparable to Vero cell plaque assays [23], specific arboviruses of interest in this study were dengue, chikungunya, yellow fever, West Nile, Sindbis, and Rift Valley fever viruses. Each 10-μl reaction mixture contained 5 μl of 2X MyTaq master-mix (Bioline, UK), 50 mM Syto-9 dye (Life Technologies, Carlsbad, USA), and a degenerate primer mix (Table 1). Cycling and HRM analysis was done in a Rotor-Gene Q real-time PCR thermo-cycler (Qiagen, Hilden Germany) using conditions described by Villinger et al. [23]. Dengue virus serotype 2 and Sindbis virus cDNA served as *Flavivirus* and *Alphavirus* positive controls, respectively, and molecular grade PCR water as the negative control. High (10^−2^) and low (10^−6^) viral titre positive controls from extraction and cDNA synthesis were included in the runs for both dengue and Sindbis viruses.

**Table 1:** Primers used for blood-meal and arboviral identification.

Target gene	Primer name	Primer sequence (5’– 3’)	Product size (bp)	References
**Vertebrate 16S**	Vert 16S F	GAGAAGACCCTRTGGARCTT	250	[25]
	Vert 16S R	CGCTGTTATCCCTAGGGTA		
**Vertebrate cyt *b***	Cytb F	CCCCTCAGAATGATATTTGTCCTCA	310	[27]
	Cytb R	CATCCAACATCTCAGCATGATGAAA		
***Alphavirus* NS4**	Vir 2052 F	TGGCGCTATGATGAAATCTGGAATGTT	150	[28]
	Vir 2052 R	TACGATGTTGTCGTCGCCGATGAA		
***Flavivirus* NS5**	Flavi JV2a F	AGYMGHGCCATHTGGTWCATGTGG	150	[23]
	Flavi JV2b F	AGCCGYGCCATHTGGTATATGTGG		
	Flavi JV2c F	AGYCGMGCAATHTGGTACATGTGG		
	Flavi JV2d F	AGTAGAGCTATATGGTACATGTGG		
	Flavi JV2a R	GTRTCCCADCCDGCDGTRTCATC		
	Flavi JV2b R	GTRTCCCAKCCWGCTGTGTCGTC		
***Flavivirus* NS5**	1NS5F	GCATCTAYAWCAYNATGGG	930	[29]
	1NS5R	CCANACNYNRTTCCANAC		
	2NS5F	GCNATNTGGTWYATGTGG		
	2NS5R	CATRTCTTCNGTNGTCATCC		
***Alphavirus* NS1**	m2w	YAGAGCDTTTTCGCAYSTRGCHW	320	[30]
	m2w2	TGYCCNVTGMDNWSYVCNGARGAYCC		
	cm3w	ACATRAANKGNGTNGTRTCRAANCCDAYCC		

All positive samples from the multiplex PCR-HRM, identified by visual inspection of the HRM profiles on the Rotor-Gene Q software 2.1.0, were selected for genus-specific (single-plex) amplification using the same reaction mixtures and cycling conditions as outlined above. Representative positive samples from the single-plex runs were selected and prepared for sequencing using the Exo 1-rSAP combination (Biolabs, UK). Bi-directional sequencing was outsourced at Macrogen (The Netherlands). Sequence chromatograms were inspected, edited, and aligned using Geneious Prime version 2019.0.4 software (Biomatters, New Zealand). The resulting sequence contigs were used in nucleotide BLAST searches against the GenBank database (www.ncbi.nlm.nih.gov) to identity the closest sequence matches.

**Fig 2:**
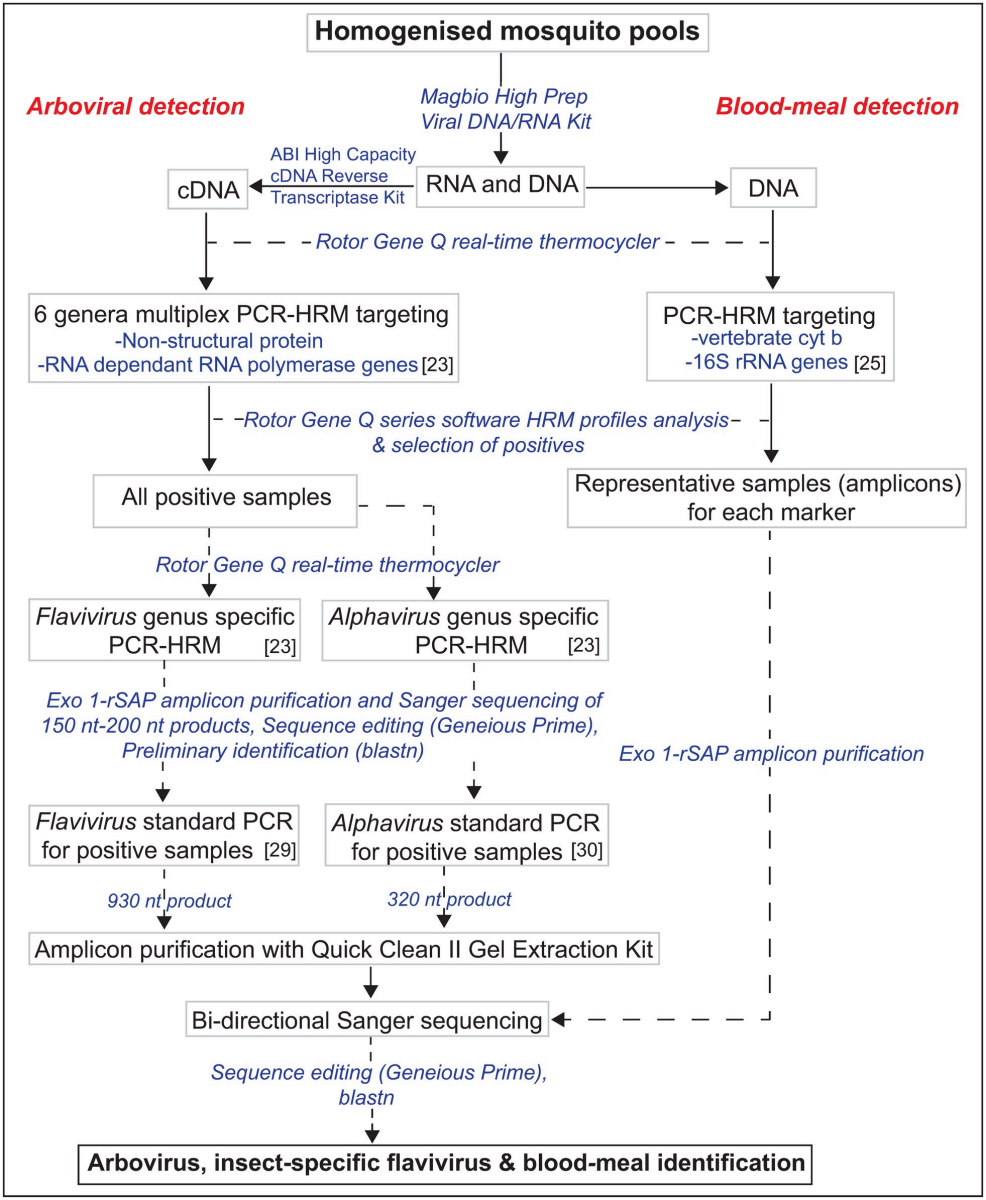
Flowchart showing the molecular detection of arboviruses, insect-specific flaviviruses and determination of blood-meal sources of blood-fed mosquitoes.

To generate a longer fragment of 900 nt for flaviviruses, positive samples were re-amplified using nested conventional PCR targeting the non-structural protein 5 (NS5) gene [29] (Table 1). The 20-μl primary reaction mix contained 4 μl 5X HOT FIREPol^®^ EvaGreen^®^ qPCR Mix (Solis BioDyne, Estonia), 10 pmoles of each primer and 1 μl of the template. For the nested amplifications, 1 μl of the first-round PCR product was used as template. Thermal cycling conditions for first and second round PCR comprised an initial hot start step of 95°C for 15 minutes followed by denaturation at 94°C for 60 secs, annealing for 40 secs, and extension at 72°C for 2 min, with a final extension at 72°C for 5 min. Annealing temperature and cycle number for first and second round PCR were 54°C and 40 cycles, and 60°C and 35 cycles, respectively. The DNA (no-RT controls) of all samples positive for flaviviruses were screened using the same methods described above for detection of flaviviruses to rule out non-specific amplification of integrated viral elements in the mosquito genome. Fig 2 illustrates the steps taken from total nucleic acid extraction to identification of blood-meal sources and detection of viruses.

For alphaviruses, to generate a longer 320-nt fragment, we used a conventional, hemi-nested PCR targeting the non-specific protein 1 (NS1) gene using primers described before [30]. The 10-μl first-round reaction mixtures contained 2 μl 5X HOT FIREPol^®^ EvaGreen^®^ qPCR Mix (Solis BioDyne, Estonia), 10 pmoles of each primer and 1 μl of the template. In the second round of amplification, 1.25 μl of the product was used as a template in a 20 μl mixture. The cycling conditions were as follows: An initial hot start step of 95°C for 15 minutes followed by 45 cycles of 94°C for 20 secs, 50°C for 30 secs, and 72°C for 30 secs, and a final extension at 72°C for 5 min. The same conditions were used for the second round except that the annealing was at 48°C. Positive controls were included in each run as above. Amplicons were visualized by gel electrophoresis. Positives were then purified for sequencing, resulting sequences edited and then identity confirmed as described before.

### Phylogenetic analysis, calculation of infection rates, and statistical analysis

A maximum likelihood phylogeny of the detected *Flavivirus* NS5 gene sequences was constructed using PHyML v. 3.0 [31]. The phylogeny employed the Akaike information criterion [32] for automatic model selection and tree topologies were estimated using nearest neighbour interchange improvements over 1000 bootstrap replicates. The phylogenetic trees were visualized using FigTree v.1.4.2 [33]. To estimate the infection rates (IRs), maximal likelihood estimates were calculated using the PooledInfRate, version 4.0 Microsoft Excel^®^ Add-In, and expressed per 100 (%) mosquitoes tested [34]. Logistic regression analysis was performed in R^®^ version 3.5.3 to test the association between sampling sites, mosquito sex, and season (predictor variables), and a mosquito pool testing positive for ISFs (outcome variable). The odds ratios (OR), 95% confidence intervals (CI), and *p*-values were computed, and a *p*-value less than 0.05 was considered statistically significant.

## Results

### Mosquito abundance and species diversity

A total of 6,848 mosquitoes were collected and assembled into 545 pools (≤25 individuals/pool) (Table 2). The 2019 long rainy season collections accounted for 89.08% (n = 6,100) of the total catch, while the 2018 short rainy season (pilot) made up the remainder (10.92%; n = 748). The collection comprised 21 mosquito species from three genera (Table 2), inclusive of 38 blood-fed specimens which were processed individually. The most abundant mosquitoes were from the genus *Culex*, contributing 59.49% (n = 4,074) of the total catch, followed by 39.66% (n = 2,716) *Aedes* and 0.85% (n = 58) *Anopheles* mosquitoes. The most abundant species were *Cx*. *pipiens* s.l. (n = 3,130) and *Ae*. *aegypti* (n = 2,661), translating to 45.71% and 38.86% of the total catch, respectively.

**Table 2:** Summary table of mosquitoes caught at the sampling sites during the long and short rainy seasons in western Kenya.

	Busia RH	Butula MH	Bungoma RH	Lugulu MH	Mukumu MH	Matungu SCH	Lubao LM	Funyula LM	Angurai LM	Chwele LM	Kimilili LM	Total
**Short rainy season (October–December 2018)**												
*Aedes aegypti*	75	84	79	48	62	43	27	-		1	1	420
*Aedes metallicus*	-	1	-	-	-	-	-	-	-	-	-	1
*Aedes* sp.	-	-	-	-	-	-	11	-		2		13
*Anopheles funestus*	-	-	-	-	-	-	1	-	-	-	-	1
*Anopheles gambiae*	-	-	-	-	-	-	5	-	-	-	1	6
*Anopheles squamosus*	-	-	-	-	-	-	1	-	-	-	-	1
*Culex annulioris*	-	-	-	-	-	-	9	-	-	-	-	9
*Culex cinerellus*	-	5	-	-	-	1	30	-	-	-	-	36
*Culex pipiens* s.l.	2	58	56	27	14	10	16	-	-	3	1	187
*Culex* sp.	-	-	-	-	-	-	-	-	4	-	-	4
*Culex vansomereni*	-	2	0	2	7	-	4	-	-	-	-	15
*Culex zombaensis*	-	40	3	4	8	-	-	-	-	-	-	55
**Subtotal**	77	190	138	81	91	54	104	-	4	6	3	**748**
**Long rainy season (May–June 2019)**												
*Aedes aegypti*	762	240	157	582	307	119	30	44	-	-	-	2,241
*Aedes africanus*	-	9	-	2	-	-	-	-	-	-	-	11
*Aedes hirsutus*	1	1	-	-	-	-	-	-	-	-	-	2
*Aedes mcintoshi*	-	-	-	-	-	1	-	8	-	-	-	9
*Aedes metallicus*	-	-	-	-	4	3	5	-	-	-	-	12
*Aedes simpsoni*	-	-	-	-	3	2	-	-	-	-	-	5
*Aedes tricholabis*	-	-	-	2	-	-	-	-	-	-	-	2
*Anopheles coustani*	-	-	-	-	-	-	3	-	-	-	-	3
*Anopheles funestus*	-	1	-	-	-	1	7	-	-	-	-	9
*Anopheles gambiae*	1	16	3	-	1	14	3	-	-	-	-	38
*Culex annulioris*	-	5	-	2	10	1	7	-	-	-	-	25
*Culex cinerellus*	-	-	-	-	-	-	5	-	-	-	-	5
*Culex cinereus*	-	11	1	-	2	3	-	-	-	-	-	17
*Culex pipiens* s.l.	255	129	646	1,273	269	307	63	1	-	-	-	2,943
*Culex poicilipes*	-	-	-	-	1	-	-	-	-	-	-	1
*Culex rubinotus*	-	5	-	-	-	-	-	-	-	-	-	5
*Culex tigripes*	1	-	-	18	-	-	1	-	-	-	-	20
*Culex univittatus*	1	5	-	-	-	-	3	-	-	-	-	9
*Culex vansomereni*	-	3	1	8	2	5	2	-	-	-	-	21
*Culex zombaensis*	114	27	119	202	85	165	10	-	-	-	-	722
**Subtotal**	1,135	452	927	2,089	684	621	139	53	-	-	-	**6,100**
**Total mosquitoes captured (short and long rainy seasons)**												**6,848**

RH = Referral hospital; MH = Missionary hospital; SCH = sub-County hospital; LM = livestock market.

A total of 6,539 mosquitoes were collected from the selected six hospitals, 631 and 5,908 during the short and long rainy seasons, respectively. Among these, the mosquito abundance was highest at Lugulu Missionary (n = 2,170), followed by Busia Referral (n = 1,212), Bungoma Referral (n = 1,065), Mukumu Missionary (n = 775), Matungu sub-County (n = 675), and Butula Missionary (n = 642) hospitals (Table 2).

BG sentinel collections consisted mostly of *Aedes* spp., of which *Ae*. *aegypti* was the dominant species, comprising 38.29% (n = 2,622) of the total catch; very few specimens of the other six *Aedes* spp. were collected (Table 2). CDC light trap collections were dominated by *Cx*. *pipiens* s.l., which accounted for 45.02% (n = 3,083) of the total catch. There was also a significant number (n = 777) of *Cx*. *zombaensis*. Overall, 309 mosquitoes were collected at LMs: 117 during the short rainy season (pilot) and 192 during the long rainy season. *Aedes aegypti* (33.33%; n = 103) was the most abundant species collected at LMs, followed by *Cx*. *pipiens* s.l. (27.18%; n = 84) (Table 2).

Of particular note were the higher total night catches of *Cx*. *pipiens* s.l. mosquitoes in the vicinity of several sewage tanks that were not covered at Lugulu Missionary (n = 1,300) and Bungoma Referral (n = 702) hospitals, compared to the other four sites where these environmental features were absent. Atypically, during the short rainy season more *Cx*. *pipiens* s.l. mosquitoes were captured at Butula Missionary Hospital compared to Lugulu Missionary and Bungoma Referral hospitals. This could be explained by the presence of unused pit latrines at Butula Missionary Hospital, and during the short rainy season their effect on *Cx*. *pipiens* s.l. abundance is comparable to open sewage tanks. In total more *Ae*. *aegypti* mosquitoes were also collected during the day at Busia Referral Hospital (n = 837) and Lugulu Missionary Hospital (n = 630), where there was a combination of huge piles of disused vehicle tyres and tall grasses, compared to the other sites where such features were less pronounced. In contrast, in the short rainy season more *Ae*. *aegypti* mosquitoes were captured at Butula Missionary, Bungoma Referral and Mukumu Missionary hospitals compared to Busia Referral and Lugulu Missionary hospitals. This could be attributed to the fact that the above mentioned favorable environmental features for *Ae*. *aegypti* proliferation were more pronounced at the former three hospitals during the short rainy season compared to the other sites.

### Blood-meal analysis

**Fig 3:**
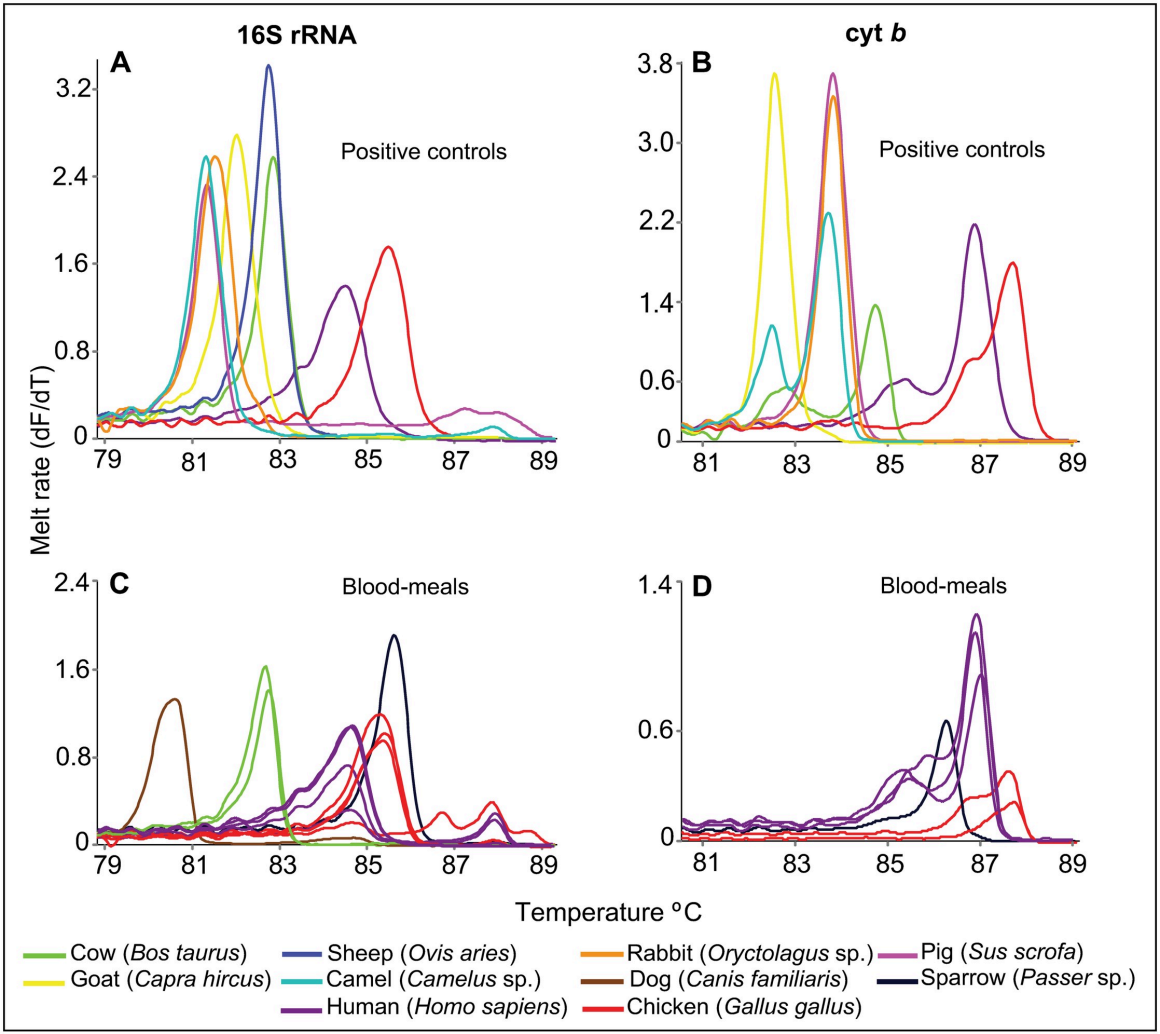
Melt rate profiles of resolved blood-meal sources from mosquitoes sampled at selected hospitals in Busia, Bungoma, and Kakamega counties. Each panel is composed of mixed curves from several controls and blood-meal samples. Amplification of (A) 16S rRNA and (B) cyt *b* markers in positive controls. Amplification of (C) 16S rRNA and (D) cyt *b* markers in positive samples.

Of the 38 blood-fed individual mosquitoes, 35 were *Cx*. *pipiens* s.l., two were *Ae*. *aegypti*, and one was *An*. *gambiae*. The blood-meals were from five vertebrate species: human, cattle, dog, chicken, and sparrow (Table 3). Two blood-meals (from one *An*. *gambiae* and one *Cx*. *pipiens* s.l.) could not be resolved by amplification with either cyt *b* or 16S rRNA markers (Table 3). For confirmation of human blood meals our vertebrate 16S rRNA gene sequences (GenBank accessions MT012144-MT012144) all shared 100% identity with human 16S rRNA sequence MK248422 in GenBank. The human vertebrate cyt *b* gene sequences (GenBank accessions MT019210-MT019216) all shared 100% identity with reference human cyt *b* sequences (e.g., GenBank accession MW389273). Our cattle vertebrate 16S rRNA sequences (GenBank accessions MT012262-MT012263) all shared 100% identity with reference cattle 16S rRNA sequence MN714195 in GenBank. Our chicken vertebrate 16S rRNA gene sequences (GenBank accessions MT012140-MT012142) all shared 100% identity with a reference chicken 16S rRNA sequence (GenBank accession MN013407). The chicken vertebrate cyt *b* sequence (GenBank accession MT019209) also shared 100% identity with a reference cyt *b* chicken sequence (GenBank accession KX512321). While positive controls for dog and sparrow were not available, confirmation of dog blood-meal source was based on the shared 98.2% identity between our 16S rRNA sequence (GenBank accession MT012139) and a reference GenBank sequence (accession MN181404). Similarly, the sparrow vertebrate 16S rRNA sequence from this study (GenBank accession MT012844) shared 95% identity with a reference sequence (GenBank accession KT895996), while the cyt *b* sequence (GenBank accession MT019217) shared 100% identity with a reference sparrow cyt *b* sequence (GenBank accession AF230908). The rather low sparrow 16S rRNA percentage identity was compensated for by amplification in the cyt *b* marker. The melt rate profiles of the samples and the positive controls are shown in Fig 3. Non-amplification in one of the markers was resolved by amplification in the other marker. Most of the blood-fed mosquitoes were caught at Matungu sub-County Hospital (Table 3).

**Table 3:** Number of blood-meal sources of mosquitoes sampled at hospitals in Busia, Bungoma and Kakamega counties.

Sampling site	Species	Human	Cattle	Dog	Chicken	Sparrow	ND^a^	Total
Bungoma RH	*Culex pipiens* s.l.	5	0	1	1	0	0	7
Busia RH	*Culex pipiens* s.l.	7	0	0	0	0	0	7
	*Aedes aegypti*	1	0	0	0	0	0	1
Butula MH	*Culex pipiens* s.l.	1	0	0	1	0	0	2
Lugulu MH	*Culex pipiens* s.l.	0	1	0	0	1	0	2
Matungu SCH	*Anopheles gambiae*	0	0	0	0	0	1	2
	*Culex pipiens* s.l.	10	0	0	1	0	1	12
Mukumu MH	*Culex pipiens* s.l.	1	0	0	4	0	0	5
	*Aedes aegypti*	0	1	0	0	0	0	1
Total blood meals		25	2	1	7	1	2	**38**

RH = Referral hospital; MH = Missionary hospital; SCH = sub-County hospital.

^a^ND: Not determined by the two markers.

### Viruses detected

While the mosquito pools analyzed were negative for most of the human pathogenic arboviruses endemic in Kenya, a single unique *Culex poicilipes* female sampled at Mukumu Missionary Hospital (Kakamega County) was positive for Sindbis virus (GenBank accession MT019267). The NS1 sequence of our Sindbis virus strain varied from that of the strain used as a positive control at twelve nucleotide position, making it unlikely that it was a false positive due to cross contamination (S1 Fig). On the other hand, it showed highest similarity (99.6%) with a Sindbis strain detected in a *Cx*. *pipiens* mosquito in Kenya (MK510862) [35].

**Fig 4:**
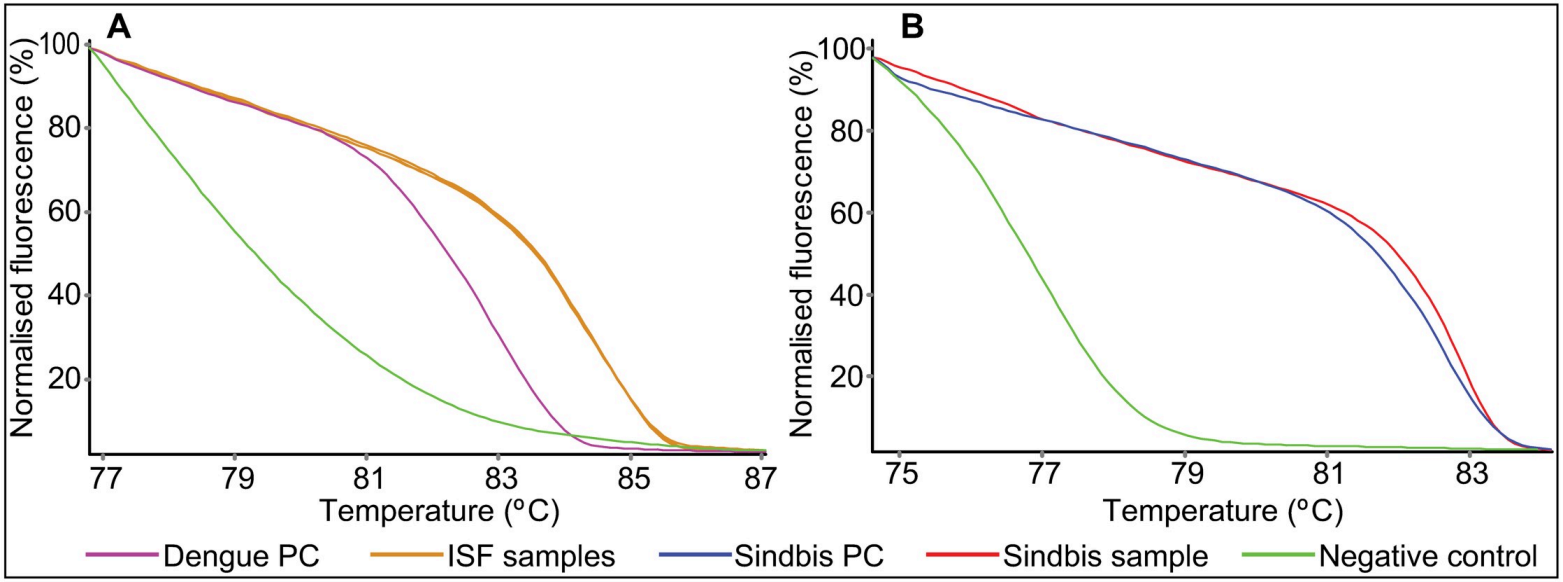
High-resolution melting profiles of mosquito pools with (A) insect-specific flavivirus and (B) Sindbis virus. PC = positive control.

A total of 49 mosquito pools were positive for ISFs, among which 30 pools were positive for cell fusing agent virus (CFAV), 11 for Aedes flavivirus (AeFV), and eight for Culex flavivirus (CxFV) (Fig 4). Nucleotide sequence identities of the NS5 gene region ranged from 98.3–100% for CFAV, 98.6–99.6% for AeFV, and 98.2–99.9% for CxFV characterized in this study with those in the Genbank database. None of the ISF-positive samples amplified using their DNA (no-RT controls), illustrating that the amplification observed was due to identified ISFs, not endogenous viral elements that may be integrated into the mosquito genome. Culex flavivirus positive mosquito pools were all comprised of *Cx*. *pipiens* s.l. mosquitoes, except for one *Culex annulioris*. All 38 fed specimens were negative for both ISFs and pathogenic arboviruses.

The overall maximum likelihood estimates of IRs for sampled *Ae*. *aegypti* with ISFs were 1.27% (95% CI = 0.87%-1.78%) for CFAV infection, and 0.43% (95% CI = 0.23%-0.74%) for AeFV. The overall IR estimate for *Cx*. *pipiens* s.l. with CxFV was 0.23% (95% CI = 0.1%-0.45%) (S1 Table). The odds of *Ae*. *aegypti* testing positive for ISFs (AeFV and CFAV) were significantly higher in Bungoma (OR = 2.53, 95% CI = 1.18–5.72, *p* = 0.02) and Kakamega (OR = 2.70, 95% CI = 1.18–6.36; *p* = 0.02) compared to Busia (Fig 1; S2 Table). For CFAV alone, the odds for *Ae*. *aegypti* to be infected were similarly higher in Bungoma (OR = 3.99, 95% CI = 1.65–11.10, *p* = 0.004) than in Busia, while there was no significant difference between sites in Kakamega and Busia. The odds of *Ae*. *aegypti* being infected with AeFV, and *Cx*. *pipiens* s.l. with CxFV, were not significantly different in the three counties (S2 Table). The odds of a mosquito being infected with ISFs was not significantly different between LMs and hospitals (OR = 0.41, 95% CI = 0.18–1.20), *p* = 0.06 (S2 Table). Both female and male pools of *Ae*. *aegypti* were positive for CFAV and AeFV, but only female *Culex* were positive for CxFV. However, the odds of *Ae*. *aegypti* being positive for ISFs (CFAV and AeFV) were not significantly different between the two sexes (S2 Table). The odds for ISFs (CFAV and AeFV) infection of *Ae*. *aegypti* were not significantly different between the two rainy seasons. Logistic regression analysis for CxFV in *Cx*. *pipiens* s.l. for seasonality and sex was not performed due to insufficient data.

**Fig 5:**
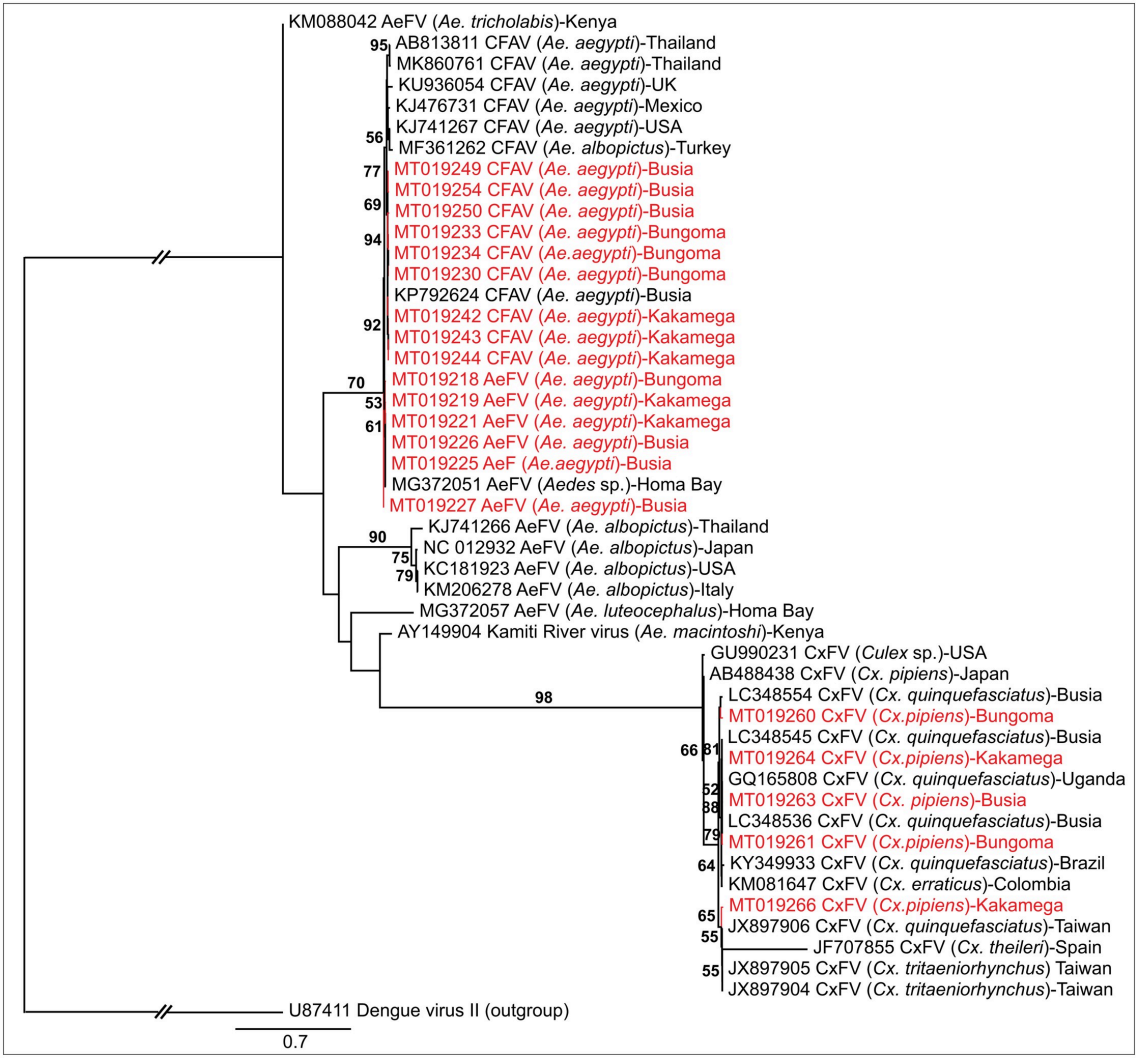
Maximum likelihood phylogeny of flaviviruses inferred from 48 aligned 900-nt segments of the NS5 gene. Taxon names include GenBank accession numbers, isolation source, and country of origin. Sequences from this study are in red. Bootstrap values at the nodes are of percentage agreement among 1,000 replicates. The branch length scale represents substitutions per site. The gaps to the outgroup represents 2.1 substitutions per site.

The CFAV NS5 (GenBank accessions MT019229-MT019258) gene sequences clustered according to county, with those from Busia being closely related to CFAV NS5 gene sequences detected previously in Busia (GenBank accession KP792624). Aedes flavivirus NS5 gene sequences from this study were related to those from Homa Bay (GenBank accession MG372051) [36] (Fig 5). One of the CxFV NS5 sequences from Kakamega (GenBank accession MT019266) clustered with two CxFV sequences from Taiwan (GenBank accessions JX897905; JX897906). Three other CxFV sequences from Kakamega (GenBank accession MT019264), Bungoma (GenBank accession MT019261), and Busia (GenBank accession MT019263) were closely related to a strain from Uganda (GenBank accession GQ165808) [37] and some strains previously found in Busia (GenBank accessions LC388536; LC3885345) [38]. All ISFs sequences also clustered together according to the mosquito species from which they were detected.

### Implementation of mosquito control methods at hospitals

At all the six participating hospitals, a public health officer, medical superintendent or administration officer provided information on the measures they implement to prevent mosquitoes from proliferating and from biting patients, visitors, and staff. The use of insecticide-treated nets provided by the Public Health Department of the Ministry of Health was reported and also observed in patient wards at all the six hospitals. Three of the hospitals reported that outdoor and indoor residual insecticide spraying, also done by the Public Health Department of the Ministry of Health, had ceased 2–10 years prior to the study. The other three hospitals were still undertaking residual insecticide spraying inside patient wards and staff quarters every four to eight months with Icon^®^ insecticide as the main insecticide used. Only one of the hospitals reported that they provided topical repellant to patients, who however generally preferred not to apply them because of the odour. Only two of the hospitals had installed window screens and in one of these, the process was still ongoing. At all the institutions, grass cutting and clearing bushes were highlighted as an important tool in the control of mosquitoes. Only one institution highlighted the importance of draining water puddles, disposal of hospital waste, and rubbish, to control mosquito breeding.

## Discussion and conclusion

These findings, which raise awareness to the presence of Sindbis virus and a variety of ISFs in potential arbovirus vectors in western Kenya calls for further investigation into the epidemiology of arboviruses in the region. The trapping procedures we employed targeted blood-seeking female mosquitoes rather than those already blood-fed, hence few blood-fed mosquitoes were collected. Mosquitoes were trapped mostly outdoors, therefore fewer *Anopheles* spp. were collected for both day and night trapping, in comparison to the more abundant *Cx*. *pipiens* s.l. and *Ae*. *aegypti*. The common malaria vectors in the study region, *Anopheles gambiae* sensu stricto, are known to be highly arthropophilic and endophilic [13]. This is in comparison to *Cx*. *pipiens* s.l., which is both endophilic and exophilic but mostly ornithorphilic [39], and *Ae*. *aegypti*, which is exophilic and anthropophilic [40].

To the best of our knowledge, this is the first report of a study screening for viral nucleic acids in mosquitoes trapped at hospitals and LMs in Kenya. Most similar studies focus on homesteads [38], peri-domestic sites [41], and/or human-wildlife interfaces [35]. The hospitals were generally representative of urban settings, which tend to achieve high mosquito catches and are important for assessing urban transmission of arboviruses. This is of epidemiological importance because patients who are arboviral carriers visiting these hospitals that have large catchments in the area can set off a transmission chain in the neighborhood through capable mosquito vectors. Sampling urban or hospital settings cannot be used to trace sylvatic to urban spillover, which happens at wildlife/forest-human interfaces. However, an exception may be at hospitals like Mukumu Missionary and Kakamega Referral (excluded from the sampling), which see patients coming from distant areas of the county, including the edge of the Kakamega forest, as referral patients from smaller health facilities. In contrast, trapping at LMs can be used to assess potential zoo-prophylactic/potentiation effects on mosquito abundance. However, the open nature of the markets results in less habitat and resting sites for mosquitoes, thereby limiting catches. Furthermore, the poor security at the open markets leaves trapping equipment susceptible to theft.

As expected, the mosquito abundance and species diversity in this study coincided with the presence of favourable habitats. *Aedes aegypti* mosquitoes are known to be highly anthropophilic and their larvae breed where there are tall grasses and artificial water stagnation [42]. For *Cx*. *pipiens* s.l., open septic tanks and pit latrines are favourable habitats for their breeding [43, 44]. Therefore, proper management of these habitats in urban and peri-urban settings will go a long way in preventing arboviral transmission and outbreaks.

The detection of Sindbis virus highlights its occurrence even in regions where outbreaks have not been reported. Sindbis virus, first isolated from mosquitoes in Egypt [45], circulates between birds and *Culex* spp. mosquitoes, with humans acting as dead-end hosts [46]. The virus causes rash, febrile illness, myalgia, and arthralgia. In Kenya, two acute cases of Sindbis virus were detected by inoculation on Vero cell lines and RT-PCR in febrile patients from Mfangano islands of Lake Victoria [47], and seroprevalence studies in other Kenyan regions have shown exposure in local populations [48, 49]. Clinical cases may be masked by other febrile illnesses, such as malaria in Kenya, and may therefore go unnoticed due to lack of awareness and inadequate diagnostic capabilities in health institutions [12, 50, 51].

Birds are known to be amplifying hosts of Sindbis and West Nile viruses [4, 52]; hence, the detection of a *Cx*. *pipiens* s.l. mosquito that had fed on a sparrow (*Passer* sp.) suggests risk of transmission of Sindbis virus from birds to humans. In Kenya, Sindbis virus has been detected in *Culex* mosquitoes [35, 53]. It is endemic in South Africa and northern Europe [4] where outbreaks have been reported. Several *Culex* spp. are vectors of the virus in different parts of the world, with *Culex univittatus* and *Cx*. *pipiens/Culex torrentium* thought to be the main vectors [54, 55]. Increased IRs in the chief vectors in northern Europe have been found to be a predictor of Sindbis related rash and arthralgia outbreaks [46].

The detection of ISFs is not likely to warrant public health concern since they have not been shown to infect or grow in vertebrate cells [56]. Previous studies in Kenya have detected CFAV, AeFV, and CxFV in mosquitoes mostly around the lake shores [23, 36, 38]. In contrast, our study reports significantly higher odds of *Ae*. *aegypti* infection by ISFs in Bungoma and Kakamega, which are more inland compared to Busia, which is closer to Lake Victoria. The local ecology has been shown to have a pronounced impact not only on mosquito abundance but also their viral infection status [57]. Higher CxFV infection has, for example, been reported in sites with dense housing, compared to urban open spaces in Chicago, USA [58]. Busia County is closer to the shores of Lake Victoria with a distinct ecology from that of Bungoma and Kakamega. While we found no significant difference between male and female mosquitoes in terms of probability of being infected with ISFs, most previous studies suggest higher infection rates in females, though these results may be biased due to low numbers of male mosquitoes collected, and in some cases males are not processed for viral detection [38]. The detection of ISFs in male mosquitoes emphasises the occurrence of vertical and venereal transmission of ISFs [36, 59]. Seasonality did not seem to have an effect on ISF positivity in this study, which could be due to the similarity of environmental variables during the short and long rainy seasons. Therefore, in the future it would be important to sample also during the dry season. In Houston, USA, virus-infected mosquito pools were detected only in the cooler months, compared to the warmer months, showing an effect of seasonality [60].

There has been growing interest in the possibility of using ISFs to interfere with the acquisition and transmission of pathogenic arboviruses [59]. Studies have shown that West Nile virus growth rate was lower in cell cultures co-infected with CxFV, compared to those not co-infected, and mosquito dissemination rates were lower in persistently-infected *Cx*. *pipiens* s.l. colonies, compared to mosquitoes not infected with CxFV [61]. Other studies have shown *in vitro* interference by CFAV on Zika virus growth [62]. However, it should be noted that the interference effect of ISFs on arboviral transmission may be specific to experimentally investigated arbovirus/ISF pairs and cannot be extrapolated to all the arboviruses and ISFs. As the high analytical sensitivity of the PCR-HRM test we employed in this study has been demonstrated [23], the disparity between arboviral and ISFs detection in this study may be due to the very low infections rates of mosquitoes by arboviruses given that sampling was performed during non-epidemic periods [63]. Seroprevalence studies in this region have reported the presence of antibodies to chikungunya, West Nile, yellow fever, and dengue viruses in the human population signifying undetected circulation [64, 65]. The clustering of ISF sequences within mosquito species shows that they are relatively conserved within mosquito species across geographical divides.

Due to logistical challenges, the number of trap days and nights were not uniform across the two rainy seasons (short and long) and the sampling sites (hospitals and LMs), limiting statistical comparisons of mosquito abundance. A more extensive sampling exercise over several seasons may lead to the detection of more pathogenic arboviruses as the IR in mosquitoes is usually very low during inter-epidemic periods [63]. Despite the methodological limitations of arboviral detection in this study, we hypothesize that the relatively high IRs with ISFs may be modulating the transmission and occurrence of arboviral outbreaks in non-endemic areas in Kenya. While the transmission-blocking potential of ISFs has been studied in the lab, it is important for future studies to compare the IRs of ISFs in our study site to those from arboviral endemic areas, such as the coast and north-eastern Kenya, to assess this effect in a field setting. This study highlights the presence of Sindbis, a pathogenic arbovirus, and ISFs in mosquitoes from western Kenya. While this entails risk of transmission to humans, it also calls for further investigation of the role of ISFs in the transmission dynamics of arboviruses.

## Supporting information

S1 Fig Alignment of non-specific protein 1 (NS1) gene segments of the Sindbis virus positive control (KY616986) used in this study and the Sindbis virus positive sample (MT019267). The green bar shows consensus between the two segments wherein the gaps indicate points of disagreements. Colour code of nucleotides are depicted as Green = Uracil; Red = Adenine; Blue = Cytosine; Yellow = Guanine.
(TIF)

S1 Table Details of mosquito pools positive for insect-specific flaviviruses^a^. (DOCX)

S2 Table Logistic regression model with County, mosquito sex, season and site use as independent variables and odds of being infected with insect-specific flaviviruses^a^. (DOCX)

S1 File A sample of the questionnaire used to collect information about mosquito control at hospitals in western Kenya. (DOCX)

## Abbreviations

AeFV: Aedes flavivirus
CFAV: cell fusing agent virus
CxFV: Culex flavivirus
cyt *b*: cytochrome b
*icipe*: International Centre of Insect Physiology and Ecology
ILRI: International Livestock Research Institute
IR: infection rate
IREC: Institutional Research Ethics Committee
ISF: insect specific flavivirus
LM: livestock market
MH: missionary hospital
ML-EID: Martin Lüscher Emerging Infectious Disease
NACOSTI: National Commission for Science, Technology and Innovation
NS1: non-specific protein 1
NS5: non-structural protein 5
PCR-HRM: polymerase chain reaction-high resolution melting analysis
RH: referral hospital
RT-PCR: reverse transcriptase polymerase chain reaction
SCH: sub-County hospital
