# Supplementary figures and images for: A survey of mosquito-borne and insect-specific viruses in hospitals and livestock markets in western Kenya
Source: PLoS One. 2021 May 28;16(5):e0252369. doi: 10.1371/journal.pone.0252369 (PMC8162702; doi:10.1371/journal.pone.0252369)

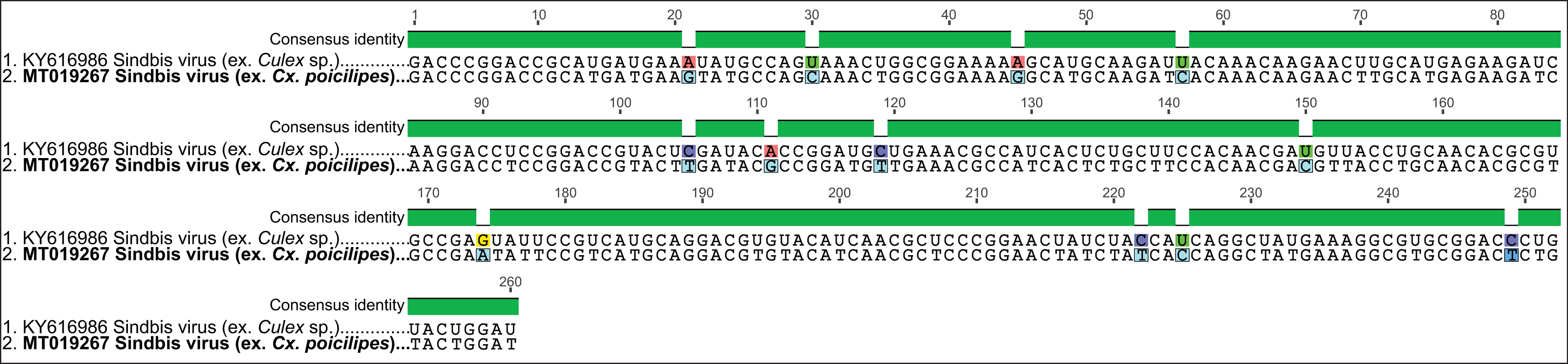

Supplement: S1 Fig — The green bar shows consensus between the two segments wherein the gaps indicate points of disagreements. Colour code of nucleotides are depicted as Green = Uracil; Red = Adenine; Blue = Cytosine; Yellow = Guanine. (TIF) [file pone.0252369.s001.tif]
