# Supplementary material for: A survey of mosquito-borne and insect-specific viruses in hospitals and livestock markets in western Kenya
Source: PLoS One. 2021 May 28;16(5):e0252369. doi: 10.1371/journal.pone.0252369 (PMC8162702; doi:10.1371/journal.pone.0252369)
Supplement: S1 Table — (DOCX) [file pone.0252369.s002.docx]

**S1 Table: Details of mosquito pools positive for insect-specific flaviviruses^a^**

| Site | Season | Species | #Mosquitoes | No. pools | CFAV (IR %; 95% CI) | AeFV (IR %; 95% CI) | CxFV (IR %; 95% CI) |
| --- | --- | --- | --- | --- | --- | --- | --- |
| **Bungoma** | County overall | ***Ae. aegypti*** | 868 | 53 | 17 (2.43); 1.48-3.83 | 1 (0.12); 0.007-0.56 | - |
|  | short rain | season total | 129 | 13 | 2 (1.67); 0.31-5 | - | - |
|  |  | females | 65 | 8 | 1 (1.62); 0.096-8.63 | - | - |
|  |  | males | 64 | 5 | 1 (1.46); 0.096-7.33 | - | - |
|  | long rain | season total | 739 | 40 | 15 (2.55); 1.51-4.14 | 1 (0.14); 0.008-0.66 | - |
|  |  | females | 329 | 18 | 7 (2.54); 1.17-5.05 | 1 (0.31); 0.02-1.5 | - |
|  |  | males | 410 | 22 | 8 (2.46); 1.16-4.79 | - | - |
| **Busia** | County overall | ***Ae. aegypti*** | 1205 | 68 | 6 (0.52); 0.21-1.08 | 4 (0.34); 0.11-0.81 | - |
|  | short rain | season total | 159 | 14 | 4 (3.20); 1.02-8.20 | - | - |
|  |  | females | 120 | 10 | 3 (3.15); 0.82-9.28 | - | - |
|  |  | males | 39 | 4 | 1 (2.71); 0.16-15.08 | - | - |
|  | long rain | season total | 1046 | 54 | 2 (0.19); 0.04-0.63 | 4 (0.39); 0.13-0.94 | - |
|  |  | females | 598 | 32 | 2 (0.34); 0.06-1.12 | 4 (0.70); 0.23-1.67 | - |
|  |  | males | 448 | 22 | - | - | - |
| **Kakamega** | County overall | ***Ae. aegypti*** | 588 | 37 | 7 (1.33); 0.59-2.64 | 6 (1.09); 0.46-2.25 |  |
|  | short rain | season total | 132 | 10 | 1 (0.75); 0.05-3.72 | 1 (0.78); 0.05-3.93 | - |
|  |  | females | 68 | 5 | 1 (1.44); 0.09-7.42 | - | - |
|  |  | males | 64 | 5 | - | 1 (1.65); 0.098-9.09 | - |
|  | long rain | season total | 456 | 27 | 6 (1.49); 0.62-3.11 | 5 (1.16); 0.44-2.54 | - |
|  |  | females | 345 | 18 | 4 (1.29); 0.43-3.16 | 3 (0.91); 0.25-2.45 | - |
|  |  | males | 111 | 9 | 2 (1.92); 0.36-6.51 | 2 (1.80); 0.36-5.84 | - |
| ***Ae. aegypti* total** |  |  | 2661 | 158 | 30 (1.27); 0.87-1.78 | 11 (0.43); 0.23-0.74 | - |
| **Bungoma** | County overall | ***Cx. pipiens* s.l.** | 2006 | 108 | - | - | 5(0.26); 0.095-0.57 |
|  | short rain | season total | 87 | 8 | - | - | 1(1.16); 0.07-6.17 |
|  |  | females | 78 | 4 | - | - | 1(1.26); 0.08-6.92 |
|  |  | males | 9 | 4 | - | - | - |
|  | long rain | season total | 1919 | 100 | - | - | 4(0.21); (0.07-0.51) |
|  |  | females | 1780 | 88 | - | - | 4(0.23); 0.08-0.55 |
|  |  | males | 139 | 12 |  | - | - |
| **Busia** | County overall | ***Cx. pipiens* s.l.** | 445 | 45 |  |  | 1(0.23); 0.01-1.10 |
|  | short rain | season total | 60 | 8 | - | - | - |
|  |  | females | 40 | 4 | - | - | - |
|  |  | males | 20 | 4 | - | - | - |
|  | long rain | season total | 385 | 37 | - | - | 1(0.26); 0.02-1.28 |
|  |  | females | 348 | 28 | - | - | 1(0.29); 0.02-1.42 |
|  |  | males | 37 | 9 | - | - | - |
| **Kakamega** | County overall | ***Cx. pipiens* s.l.** | 679 | 63 |  |  | 1(0.15); 0.009-0.72 |
|  | short rain | season total | 40 | 10 | - | - | - |
|  |  | females | 35 | 7 | - | - | - |
|  |  | males | 5 | 3 | - | - | - |
|  | long rain | season total | 639 | 53 | - | - | 1(0.16); 0.009-0.76 |
|  |  | females | 592 | 44 | - | - | 1(0.17); 0.01-0.82 |
|  |  | males | 47 | 9 | - | - | - |
| ***Cx. pipiens* total** |  |  | 3130 | 216 |  |  | 7(0.23); 0.10-0.45 |

Detail of the single *Cx. annulioris* positive for CxFV not included in table

^a^CFAV = Cell fusing agent virus; CxFV = Culex flavivirus; AeFV = Aedes flavivirus; IR = Infection rate.
