# Supplementary material for: A survey of mosquito-borne and insect-specific viruses in hospitals and livestock markets in western Kenya
Source: PLoS One. 2021 May 28;16(5):e0252369. doi: 10.1371/journal.pone.0252369 (PMC8162702; doi:10.1371/journal.pone.0252369)
Supplement: S2 Table — (DOCX) [file pone.0252369.s003.docx]

**S2 Table: Logistic regression model with County, mosquito sex, season and site use as independent variables and odds of being infected with insect-specific falviviruses^a^**

| **Variable** | **Category** | **Infection rate (95% CI)** | **Odds ratio (95% CI)** | ***p*-value** |
| --- | --- | --- | --- | --- |
| *CFAV+AeFV* |  |  |  |  |
| Site | Bungoma | 2.63 (1.62-4.10) | 2.53 (1.18-5.72) | *0.02* |
|  | Kakamega | 2.74 (1.56-4.58) | 2.70 (1.18-6.36) | *0.02* |
|  | Busia | 0.89 (0.46-1.59) | Reference |  |
| Sex | Female | 2.04 (1.37-2.95) | 1.30 (0.69-2.52) | 0.43 |
|  | Male | 1.50 (0.88-2.41) | Reference |  |
| Season | Long rainy | 1.71 (1.21-2.37) | 0.77 (0.37-1.80) | 0.51 |
|  | Short rainy | 2.29 (1.07-4.42) | Reference |  |
| *CFAV* |  |  |  |  |
| Site | Bungoma | 2.43 (1.48-3.83) | 3.99 (1.65-11.10) | *0.004* |
|  | Kakamega | 1.33 (0.59-2.64) | 2.41 (0.80-7.51) | 0.12 |
|  | Busia | 0.52 (0.21-1.08) | Reference |  |
| Sex | Female | 1.33 (0.82-2.06) | 1.12 (0.54-2.40) | 0.77 |
|  | Male | 1.17 (0.64-1.99) | Reference |  |
| Season | Long rainy | 1.14 (0.75-1.68) | 0.61 (0.27-1.55) | 0.26 |
|  | Short rainy | 1.67 (0.44-2.89) | Reference |  |
| *AeFV* |  |  |  |  |
| Site | Bungoma | 0.12 (0.007-0.56) | 0.35 (0.02-2.35) | 0.34 |
|  | Kakamega | 1.09 (0.46-2.25) | 3.10 (0.88-12.15) | 0.08 |
|  | Busia | 0.34 (0.11-0.81) | Reference |  |
| *CxFV* |  |  |  |  |
| Site | Bungoma | 0.26 (0.095-0.57) | 1.11 (0.18-21.28) | 0.93 |
|  | Kakamega | 0.15 (0.009-0.72) | 0.65 (0.03-16.59) | 0.77 |
|  | Busia | 0.23 (0.01-1.10) | Reference |  |
| *CFAV*+*AeFV*+*CxFV* |  |  |  |  |
| Site use | Hospital | 0.72 (0.53-0.96) | 0.41 (0.18-1.20) | 0.06 |
|  | Livestock market | 1.79 (0.67-3.99) | Reference |  |

*P*-values below 0.05 are shown in italics

^a^CFAV = Cell fusing agent virus; AeFV = Aedes flavivirus; CxFV = Culex flavivirus
