# Supplementary material for: A survey of mosquito-borne and insect-specific viruses in hospitals and livestock markets in western Kenya
Source: PLoS One. 2021 May 28;16(5):e0252369. doi: 10.1371/journal.pone.0252369 (PMC8162702; doi:10.1371/journal.pone.0252369)
Supplement: S1 File — (DOCX) [file pone.0252369.s004.docx]

**Mosquito control questionnaire**

**Hospital**………………………………………………….... **Date**…………………………

**Name of interviewee**…………………………… **Position at hospital**……………………….

1. Do you implement any mosquito control methods at this hospital?

…………………………………………………………………………………………………

1. If **YES** can you explain what you do?

| **Control activity** | **YES/NO** | **Comments (frequency, seasonality, type of insecticide [indoor/outdoor], ITN or not, etc.** |
| --- | --- | --- |
| Bed nets |  |  |
| Insecticide spray |  |  |
| Topical repellants |  |  |
| Window screens |  |  |
| Grass cutting and bush clearing |  |  |
| Draining of water puddles |  |  |
| Disposal of rubbish and hospital waste |  |  |
| Covering disused pit latrines and sewage manholes |  |  |

Other……………………………………………………………………………………………

Comments………………………………...................................................................................................................................................................................................................................................................................................................................................................................................................................................................................................................................................................................................................................................................................................................................................................................................................................................................

1. Observations:

| **Observation** | **Pres/Abs** | **Comments** |
| --- | --- | --- |
| Disused pit latrines |  |  |
| Open sewage manholes |  |  |
| Disused dilapidated buildings |  |  |
| Piled up rubbish/waste |  |  |
| Broken down vehicles |  |  |
| Disused tyres |  |  |
| Broken down equipment |  |  |
| Water puddles |  |  |
| Tall grass and bushes |  |  |
